# The negative impact of levothyroxine treatment on urinary luteinizing hormone measurements in pediatric patients with thyroid disease

**DOI:** 10.3389/fendo.2023.1236710

**Published:** 2023-12-12

**Authors:** And Demir, Ece Böber, Sükran Darcan, Adem Aydın, Ulf-Håkan Stenman, Atilla Büyükgebiz, Matti Hero

**Affiliations:** ^1^ Pediatric Research Center, New Children’s Hospital, University of Helsinki and Helsinki University Hospital, Helsinki, Finland; ^2^ Department of Pediatrics, Division of Pediatric Endocrinology, Dokuz Eylül University Faculty of Medicine, Izmir, Türkiye; ^3^ Department of Pediatrics, Division of Pediatric Endocrinology, Ege University Faculty of Medicine, Izmir, Türkiye; ^4^ Department of Pediatrics, Dokuz Eylül University Faculty of Medicine, Izmir, Türkiye; ^5^ Department of Clinical Chemistry; University of Helsinki and Helsinki University Hospital, Helsinki, Finland; ^6^ Department of Pediatrics, Division of Pediatric Endocrinology, Demiroğlu Bilim University, Istanbul, Türkiye

**Keywords:** luteinizing hormone, follicle-stimulating hormone, thyroid-stimulating hormone, urinary gonadotropins, levothyroxine

## Abstract

**Objectives:**

Previous studies suggest urinary luteinizing hormone (LH) and follicle-stimulating hormone (FSH) measurements by immunofluorometric assays (IFMA) as noninvasive alternatives to serum assays for puberty assessment. However, these studies excluded patients with other endocrine disorders and those taking medications. Besides, the recent discontinuation of IFMA manufacturing is a concern. We explored the utility of luminometric assays (LIA) for urinary gonadotropins and thyroid-stimulating hormone (TSH) determinations in euthyroid patients with thyroid pathologies.

**Methods:**

We used LIA and IFMA assays to measure serum and first-morning-voided (FMV) urine LH, FSH, and TSH concentrations in euthyroid patients with various thyroid disorders. Of the 47 euthyroid patients with normal serum TSH (S-TSH) levels, 14 were receiving levothyroxine therapy.

**Results:**

FMV total urinary LH (U-LH) concentrations correlated significantly with those measured in serum using either LIA (r=0.67, *P*<.001) or IFMA (r=0.83, *P*=.003) in patients not receiving levothyroxine treatment; however, no significant correlation could be detected in patients receiving levothyroxine regardless of the assay method (for LIA: r=0.50, *P*=.08 and IFMA r=0.44, *P*=.15). Urinary TSH (U-TSH) concentrations correlated poorly with those in serum in both the untreated and the treated groups (r=-0.13, *P*=.49, and r=-0.45, *P*=.11, respectively).

**Conclusion:**

FMV total U-LH determinations by LIA can be used to assess pubertal development in patients with thyroid pathology, provided the euthyroid patient is not on levothyroxine treatment. U-TSH measurements by LIA cannot replace invasive S-TSH measurements at least in patients with normal S-TSH levels. Further research may reveal the utility of U-TSH determinations in patients with elevated S-TSH levels.

## 1 Introduction

Prior studies have shown that ultrasensitive immunofluorometric assays (IFMA) can measure luteinizing hormone (LH) and follicle-stimulating hormone (FSH) levels in first-morning-voided (FMV) urine samples, providing a potential noninvasive alternative to serum assays for evaluating puberty and its disorders (1–13). However, these studies have consistently excluded patients with endocrinologic, nephrologic, oncologic, or neurologic disorders, or those receiving medication at the time of sampling.

Given the structural and size similarities between FSH, LH and TSH, all of which share nearly identical alpha subunits and unique beta subunits, urinary TSH testing may emerge as a noninvasive alternative. Modern sensitive assays make its detection theoretically feasible for the development of a non-invasive screening test, a capability that was lacking in the radioimmunoassays of the 1980s (14). Urinary TSH excretion tends to be elevated in patients with primary hypothyroidism (15), unless complicated by comorbidities such as nephrotic syndrome, chronic renal failure, or tubular dysfunction since the filtered TSH and hormones of similar size and structure are efficiently reabsorbed in the proximal tubules subsequent to glomerular filtration (16, 17). Hypothyroidism is a common condition, with an estimated incidence of 4.1 cases per 1,000 women annually and 0.6 cases per 1,000 men per year (18). Up to 50% of euthyroid appearing patients who have undergone radioiodine therapy or surgery may have subclinical hypothyroidism (19). While serum thyroid-stimulating hormone (TSH) levels within normal limits suggest a lower risk of progression to overt hypothyroidism, the use of urinary gonadotropin measurements to evaluate pubertal development in such patients has not been confirmed in any study in relation to a potential suppression of the hypothalamic-pituitary-gonadal (HPG) axis in both males (20) and females (21). While there are several studies that have documented the beneficial effects of T4 replacement on male gonadal function, there is a lack of similar studies in women (22). Therefore, it is important to investigate whether other endocrine diseases or treatments could affect urinary gonadotropin measurements, including in euthyroid patients with different thyroid disorders and those treated with levothyroxine.

In this study, we aimed to explore the use of noninvasive urinary gonadotropin measurements, at least in thyroid patients who maintain a euthyroid state, and determine whether being on or off T4 replacement affects this measurement. Another concern was the recent discontinuation of IFMA kits (Wallac Ltd., PerkinElmer Finland, Turku, Finland) for LH and FSH diagnostics, which have been the primary choices of test in the vast majority of studies on urinary gonadotropin determinations. Alternative assay platforms are needed, and we have analyzed different assay methods to determine gonadotropins, especially total LH immunoreactivity in urine samples. In this exploratory study, we investigated the applicability of luminometric assays (LIA) as an alternative to IFMA for measuring urinary gonadotropins in euthyroid patients with different thyroid pathologies. We also evaluated the potential utility of urinary TSH (U-TSH) measurements by LIA and IFMA as U-TSH measurements may further expand the scope of noninvasive testing.

## 2 Materials and methods

### 2.1 Patients, samples and study design

This study included outpatients with signs or symptoms of thyroid disorders, such as autoimmune thyroiditis, congenital hypothyroidism, nodular goiter, multinodular goiter, or simple diffuse goiter, who visited the outpatient clinics of Dokuz Eylül University Hospital and Ege University Hospital. Inclusion criteria required a confirmed diagnosis of thyroid disease and a euthyroid state, as confirmed by normal TSH, total T4, free T4, and T3 analyses. Exclusion criteria excluded patients with underlying diseases or hypo/hyperthyroid states, other than a pathology confined to the thyroid gland. Patients whose euthyroid state was confirmed by both clinical and biochemical criteria were included in the study, with a mean age of 13.4 ± 3.6 years. Patient information on pubertal development is summarized in **Table 1**, with 14 patients receiving levothyroxine treatment and 33 patients not receiving treatment for at least 3 months at the time of sampling. Before making a decision to include or exclude subjects, their medical history and any previous diagnosis or treatment were recorded. Physical examination was performed, including thyroid palpation and, in selected cases, thyroid ultrasonography. Pubertal development was assessed based on Tanner staging (23) and measurement of testicular volume using a Prader orchidometer. All subjects underwent routine tests to confirm their eligibility for the study, including urinalysis, complete blood count, liver function tests, kidney function tests, and thyroid function tests. TSH, total T4, free T4, and T3 analyses were used to confirm euthyroidism in all patients, including those taking levothyroxine. Patients with high TSH levels but normal T4 or free T4 were excluded to avoid compensated hypothyroidism. LIA and IFMA assays were performed in Turkey and Finland to measure LH, FSH, and TSH concentrations in serum and urine samples. Patients were instructed to collect FMV urine samples and bring them to the hospital within one hour.

**Table 1:** Patient information regarding pubertal development at the time of the study (in two on and off levothyroxine treatment subgroups).

Patient information regarding pubertal development			
	On-treatment	Off-treatment	All
	n=14	n=33	n=47
Age	13.94 (4.78)	13.17 (2.48)	13.40 (3.29)
Tanner stage – Girls (B1/2/3/4/5)	0/0/4/2/5	4/3/16/8/0	4/3/20/10/5
Tanner stage – Boys (G1/2/3/4/5)	2/0/1/0/0	2/0/0/0/0	4/0/1/0/0
Testis volume (mL)	3.00 (1.73)	2.00 (0.00)	2.60 (1.34)
Pubic hair stage (1/2/3/4/5)	2/1/2/3/6	5/3/15/10/0	7/4/17/13/6

Mean (standard deviation) values are depicted for age and testis volumes; B and G represent Tanner breast and gonadal stages, respectively.

### 2.2 Storage

Serum samples were stored at -20°C, while urine samples were stored at 4°C. To avoid U-LH degradation, urine samples were stored at +4°C for 2-4 weeks (1). The validity of the LH and FSH assays was established in a previous study, which showed that urine matrix had no effect on the results, allowing for storage at +4°C for up to 7 weeks without significant loss of LH and FSH.

### 2.3 Assays

Luminometric assays were performed to determine LH, FSH, and TSH concentrations in serum and urine using a non-isotopic immunoassay analyzer called LIA-MAT (Byk-Sangtec Diagnostica, Dietzenbach, Germany). The assays were calibrated against WHO reference preparations and precision studies were conducted to determine intra- and inter-assay variations. Intra-assay coefficients of variation (CV) for LH ranged from 2.2% to 3.5%, while intra-assay CV for FSH ranged from 2.4% to 4.7%. Inter-assay CV for LH ranged from 5.7% to 6.9%, while inter-assay CV for FSH ranged from 3.8% to 6.5%. Detection limits for LH and FSH were less than 0.4 IU/L and 0.5 IU/L, respectively. The working range for TSH was 0.33 to 100 µIU/mL, and inter-assay variability ranged from 4.7% to 12.2%. This study utilized also immunofluorometric assays (IFMA) using monoclonal antibodies (AutoDELFIA hFSH and hLH) to detect total LH and FSH in serum and urine. The assays were calibrated against WHO Second International Standards for Pituitary LH and FSH, respectively. The intra-assay CV for the LH and FSH assays were less than 2% at levels between 3 and 250 IU/L and approximately 10% at 0.3 IU/L. The inter-assay CV was less than 3% at 4-18 IU/L for both FSH and LH. The U-FSH and U-LH assays had detection limits of 0.018 and 0.015 IU/L, respectively, with intra- and inter-assay CVs ranging from 2.3% to 7.8% and 5.2% to 8.7%, respectively (1). Hormone concentrations were not corrected for variations in urinary excretion rate (1).

### 2.4 Statistical analyses

Power analysis was not conducted due to the exploratory nature of this study. We assessed normality using Kolmogorov-Smirnov and Shapiro-Wilk tests and determined the appropriate statistical test, either parametric or nonparametric, accordingly. To check for homogeneity of variances among groups, we used Levene’s test. Mann-Whitney U test was used to compare independent groups, such as patients receiving or not receiving levothyroxine treatment, based on discrepancies in hormone levels or their ratios. For paired analyses, such as investigating the differences in hormone concentrations in the same samples or different samples from the same patients, we used the Wilcoxon signed-rank test. Spearman’s rank correlation coefficient was preferred for nonparametric analysis of the correlation between urine and serum hormone concentrations and age or Tanner stage, given the results of normality tests and small sample size. A *P*-value less than 0.05 was considered statistically significant.

### 2.5 Ethical issues

The study followed the ethical guidelines for research involving human subjects as outlined in the World Medical Association’s Declaration of Helsinki of 1964, revised in 2013. Written informed consent was obtained from all parents/guardians and children aged 6 years or older and was kept on file at their respective institutions.

## 3 Results

**Figure 1:**
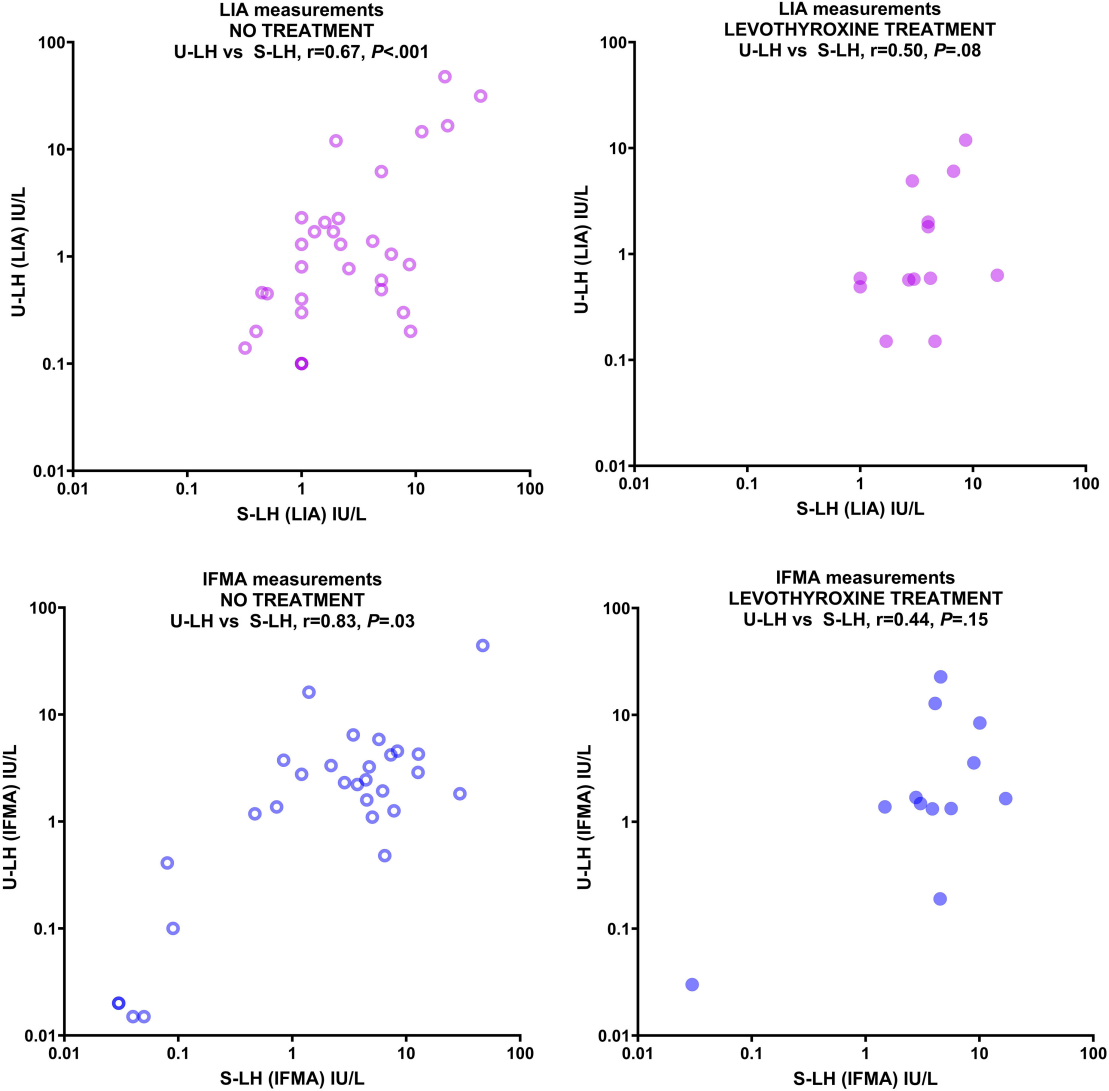
Correlation between serum and urine LH concentrations measured by LIA (upper panels) and IFMA (lower panels) in patients with various thyroid pathologies not treated with levothyroxine (left panels) and treated with levothyroxine (right panels). Concentrations are expressed in IU/L on a logarithmic scale.

Fourty-seven children or adolescents (42 girls and 5 boys, aged 0.7-18.5 yr) were included in the study. The descriptive characteristics (age, Tanner stage, pubic hair stage; and in boys, testis volume) of the levothyroxine-treated and untreated patients were not statistically different from each other (**Table 1**). Gonadotropin and TSH concentrations in serum and urine of patients with various thyroid pathologies treated and not treated with levothyroxine treatment are summarized in **Table 2**. There were no statistically significant differences between LIA and IFMA results obtained from serum and urine LH, FSH, TSH measurements in both untreated and levothyroxine-treated patients, except for U-FSH in the untreated group (**Table 2**). Serum LH concentrations measured by LIA and IFMA correlated strongly (r=0.67, *P<*.001) and very strongly (r=0.83, *P=*.003) with those in urine, respectively in patients who were not treated with levothyroxine (**Figure 1**); this correlation was absent in the treated group (r=0.50, *P=*.08 and r=0.44, *P=*.15, respectively) (**Figure 1**).

**Table 2:** Mean, S.D., and range of LH, FSH and TSH concentrations determined by LIA and IFMA in serum and urine of euthyroid patients with various thyroid pathologies receiving and not receiving levothyroxine treatment, respectively.

Descriptive statistical data regarding gonadotropin and thyrotropin concentrations					
Patients treated with levothyroxine	LIA		IFMA		*P*-value
	Mean	SD / Range	Mean	SD / Range	
S-LH (n=13)	4.68	4.12 / 1.00-16.40	5.50	4.59 / 0.03-17.00	0.50
U-LH (n=14)	2.21	3.32 / 0.40-11.90	4.16	6.42 / 0.03-22.70	0.40
S-FSH (n=13)	3.50	1.52 / 0.90-5.45	4.67	4.20 / 0.17-12.70	0.61
U-FSH (n=14)	7.47	7.65 / 0.50-25.00	3.46	5.68 / 0.03-21.40	0.06
S-TSH (n=14)	1.33	0.91 / 0.33-3.00	–		N.A.
U-TSH (n=14)	1.99	1.98 / 0.33-5.00	–		N.A.
Patients not treated with levothyroxine	LIA		IFMA		*P*-value
	Mean	SD / Range	Mean	SD / Range	
S-LH (n=31)	5.15	7.63 / 0.32-37.00	6.21	9.86 / 0.03-47.00	0.94
U-LH (n=33)	4.67	10.07 / 0.40-47.60	5.84	10.25 / 0.02-44.20	0.07
S-FSH (n=31)	3.40	2.35 / 0.70-10.0	4.19	3.38 / 0.07-13.50	0.50
U-FSH (n=33)	8.18	8.30 / 0.50-32.30	3.20	5.12 / 0.03-11.70	<.001
S-TSH (n=33)	1.93	0.83 / 0.50-3.00	–	–	N.A.
U-TSH (n=33)	1.81	1.62 / 0.33-5.50	–	–	N.A.

S.D., Standard deviation; LH, Luteinizing hormone; FSH, Follicle-stimulating hormone; TSH, Thyroid-stimulating hormone; LIA, Luminometric assay; IFMA, Immunofluorometric assays. Hormone concentrations are depicted in IU/L. FSH and LH concentrations are depicted in IU/L, and TSH concentrations in µIU/mL.

**Figure 2:**
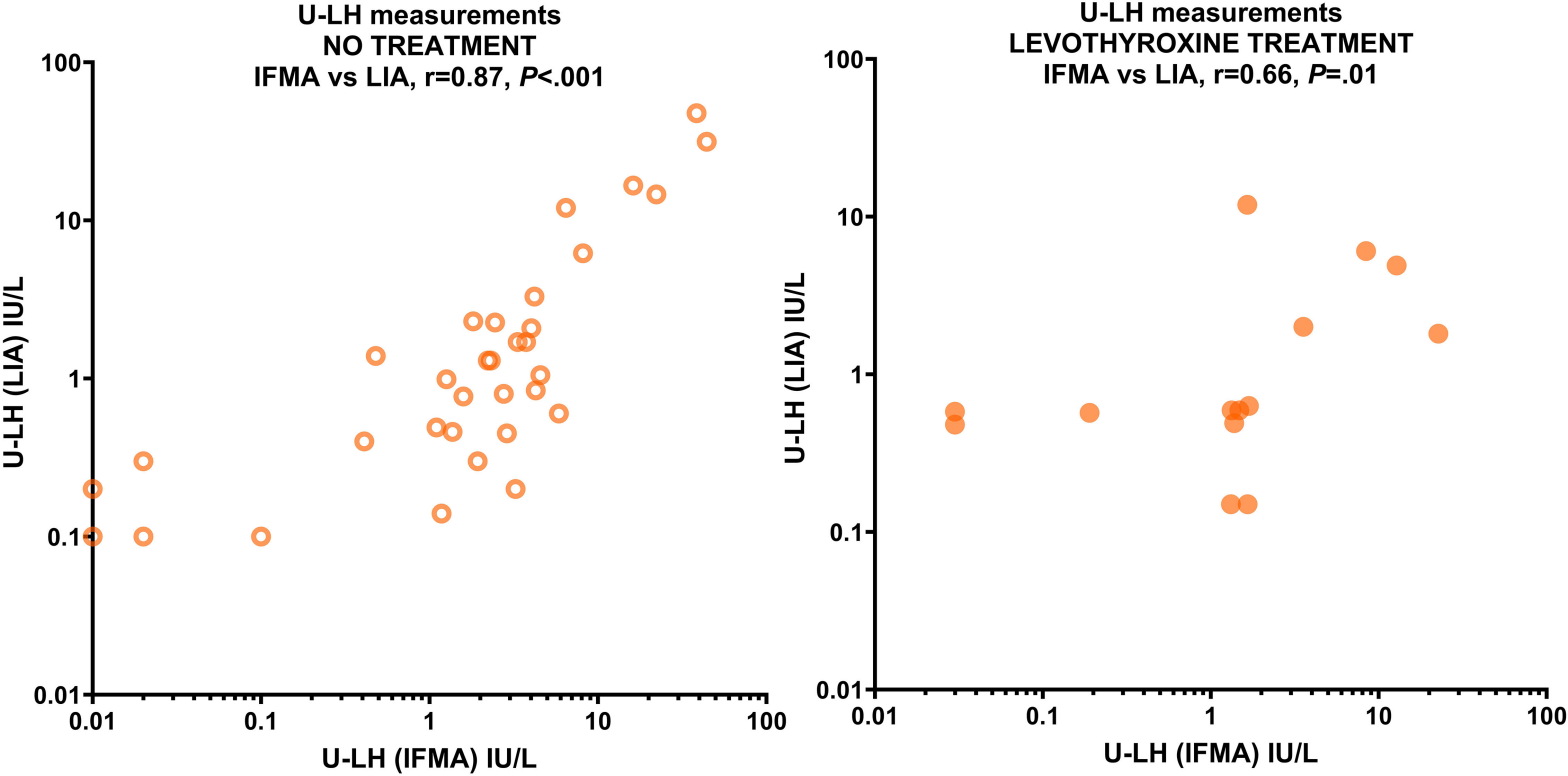
Correlation between U-LH concentrations measured by IFMA and LIA in patients with various thyroid pathologies not receiving levothyroxine treatment (left panel) and receiving levothyroxine treatment (right panel). Concentrations are expressed in IU/L on a logarithmic scale.

Because S-LH concentrations were mostly in the 1-10 IU/L range for both IFMA and LIA measurements, additional statistical analysis was performed only for this relatively more homogeneous group; however, the phenomenon described above was still evident: LH concentrations measured in serum by LIA and IFMA correlated very strongly with those in urine (r=0.84 and r=0.93, respectively; *P<*.001 for both) in patients not treated with levothyroxine; however, this correlation was absent in the treated group by both methods (r=0.28, *P=*.34 and r=0.21, *P=*.51, respectively). The U-LH concentrations measured by LIA and IFMA correlated very strongly in the untreated group (r=0.87, *P<*.001), and strongly in the treated group (r=0.66, *P=*.01) (**Figure 2**). Levothyroxine treatment had no significant effect on the correlation between S-FSH and U-FSH concentrations, nor on the correlation between the U-FSH concentrations measured by LIA and IFMA measurements (data not shown). U-TSH concentrations correlated poorly with serum concentrations in both the untreated and treated groups (r=-0.13, *P=*.49 and r=-0.45, *P=*.11, respectively). Because of the discrepancies and poor correlation between urinary gonadotropin concentrations measured by IFMA and LIA, the investigation of replacing the former method with the latter could not be completed.

## 4 Discussion

This study confirmed the strong correlation between urinary (U-LH) and serum (S-LH) concentrations of LH, with IFMA showing a stronger correlation than LIA (10). This discrepancy between the correlation levels might well be attributed to the fact that the IFMA method can detect very low prepubertal concentrations down to 0.02 IU/L, while the LIA method is unable to detect levels at below 0.1 IU/L. Our findings are consistent with previous research that pointed out the limitations of LIA in measuring urinary gonadotropins (10). Indeed, detecting changes at very low levels of LH is essential for the evaluation of prepuberty and the onset of central (biochemical) puberty. This isolated finding about the limitation of the LIA method down to 0.1 IU/L does not unequivocally establish the overall superiority of IFMA over LIA in terms of clinical utility. This is because two recent studies of ours have revealed that the onset of central puberty occurs at an FMV U-LH concentration of approximately 0.6 IU/L in girls and 0.7 IU/L in both sexes, respectively (24, 25). To compare the clinical utility of these assays in terms of parameters such as sensitivity and specificity, a ROC analysis should be conducted against a gold standard method, such as a GnRH-stimulation test or an early morning S-LH cut-off level (≤0.3 IU/L). Alternatively, to compare the technical performance of these assays, additional studies should be carried out on a larger cohort of samples featuring low, mid, and high levels of LH; subsequently, a Passing-Bablok regression analysis should be performed. The study suggests that noninvasive urinary gonadotropin measurement can be a useful tool for assessing pubertal development in euthyroid patients. However, the correlation between U-LH and S-LH concentrations was weaker in patients undergoing levothyroxine treatment, making it unsuitable for monitoring pubertal development in such cases. Levothyroxine withdrawal can be considered in children with normal T3, T4, and TSH levels after 2 years of regular treatment, and the best observation period for drug withdrawal should be 2-3 months. Therefore, U-LH determinations can be used to monitor pubertal development in euthyroid patients who have been off levothyroxine treatment for at least 3 months.

The study found that levothyroxine treatment did not negatively impact the correlation between U-LH concentrations measured by LIA and IFMA methods. However, a discordance of U-LH concentrations was observed when compared with S-LH concentrations, indicating that the discordance is not related to assay selection. Gonadotropins heterogeneity is known to affect the immunological properties of LH and thus on the immunoassay results (26). This heterogeneity affects the immunological properties of LH and may lead to the so-called “undetectable” or “invisible LH” phenomenon, which emphasizes the importance of understanding the conformational variants of LH, and from a practical perspective, highlights the need for improved standardization of gonadotropin assays (26). The same study suggests that the mechanism involving decreased reabsorption of gonadotropins with modified sialic acid residues plays a role in this discordance (26). Future research may unravel if levothyroxine treatment affects the bioactive to immunoreactive ratio of gonadotropins by inducing differences in glycosylation, molecular heterogeneity, and conflicting levels of immunoreactivity in *in-vitro* assays. Another explanation for the difference in U-LH and S-LH levels in the treated group could be the compensation of prepubertal nocturnal pulse deficits induced by hypothyroidism before levothyroxine treatment. According to Mann et al., the KNDy neurons play a significant role in connecting thyroid action to the GnRH neuronal network, and the release of pulsatile GnRH depends on the permissive action of thyroid hormone during early childhood (27).

The low bioactivity of gonadotropins in women with hypothyroidism was previously published by Tomasi et al. who reported elevated gonadotropin levels with a normal pulsatility of the gonadotropin secretion, possibly due to a decreased biological potency of the gonadotropins (28). Furthermore, treatment of hypothyroidism has been shown to reverse the partial suppression of the hypothalamic-pituitary-gonadal axis in adult women (22). Thus, an adequate levothyroxine treatment can increase the activity of LH pulses and secretion of bioactive LH into the nocturnal circulation (29–31). Nocturnal LH activity can be determined in FMV urine samples (3, 6), but not necessarily in daytime serum samples. Therefore, the correlation between FMV U-LH and daytime S-LH concentrations would be expected to be weak or absent in the late prepubertal and early-to-mid pubertal subgroup of the subjects receiving levothyroxine in this study. Because the population receiving levothyroxine treatment was not large enough to analyze among different pubertal stages, it was not possible to analyze this phenomenon in the current study; however, we hypothesize that this is the most likely explanation for the bias in the correlation between FMV U-LH and S-LH concentrations in the treatment group.

Due to the exclusion of patients with unconfirmed euthyroid states, the number of patients receiving levothyroxine treatment in the study was limited to less than half of the untreated group. This resulted in a narrower data distribution in the treated group, with the majority having S-LH concentrations between 1-10 IU/L measured by IFMA or LIA. Although the correlation between urinary gonadotropin measurements and levothyroxine treatment was poor, this was not solely due to the small number of treated patients. The non-parametric Spearman’s correlation between U-LH concentrations in the treated and untreated groups was similar and consistent with previous studies involving larger groups of patients (10). Similar results were also observed in a more homogeneous group of patients with S-LH concentrations between 1-10 IU/L measured by IFMA or LIA. A larger sample size would be advantageous to account for variations in microheterogeneity at different pubertal stages, which can be affected by sex steroids altering gonadotropin glycosylation and antibody recognition in both IFMA and LIA (32). Another limitation may be overcome by studying patients receiving similar doses of levothyroxine or by incorporating differences in levothyroxine doses in the statistical analysis (e.g., calculating adjusted correlation) since different doses of levothyroxine may alter the bioactive to immunoreactive ratio of the gonadotropins, which is an indirect index to measure differences in glycosylation that may alter the detection by monoclonal antibodies (26, 33).

The poor correlation between U-TSH and S-TSH concentrations obtained by LIA measurements indicates either that U-TSH measurements cannot be used as an alternative to invasive serum determinations in general, or that LIA is not the appropriate assay method for measuring U-TSH. In an previous study, we demonstrated the high noise-to-signal ratio of LIA assays only at levels below 5 IU/L (10). In this study, the S-TSH and U-TSH concentrations in both levothyroxine-treated and untreated patients ranged from a minimum of 0 IU/L for both to a maximum level of 5.5 IU/L and 5.0 IU/L, respectively (**Table 2**). Therefore, the results of this study should not be interpreted as a definitive failure of U-TSH determinations, but rather as an indication of either the ineffectiveness of LIA assays, or else the inadequacy of euthyroid cases for this purpose. Therefore, further research is needed to clarify the applicability of noninvasive U-TSH assays as an alternative method to serum testing in patients with high S-TSH levels. Further research can also be conducted by using alternative ultrasensitive assays to evaluate the usability of U-TSH measurements in euthyroid patients.

As reported in our recent studies, total LH immunoreactivity is derived from three different forms of LH (intact LH and its degradation products, namely the LH beta-subunit, and the 12 kD fragment of LH beta, called the core fragment) (34–36). Due to the different composition of LH isoforms and degradation products, a thorough analysis of different LH assay methods to measure the intact and total LH immunoreactivity in a larger cohort using formal power calculations is required. Such a study design with a substantially larger number of subjects would also allow for stratification of the data by pubertal stage using multivariate analysis.

We can conclude that total LH determinations by LIA in FMV urine samples can be used to evaluate pubertal development in patients with thyroid pathology as an alternative to the discontinued IFMA assays and as a general alternative to invasive serum assays, provided that the patient is in a euthyroid state and has not been on levothyroxine treatment for at least three months prior to sampling. U-TSH measurements by LIA cannot replace invasive S-TSH measurements in euthyroid cases; however, future research may clarify the clinical utility of U-TSH determinations in patients with high S-TSH levels.

## Data availability statement

The original contributions presented in the study are included in the article/supplementary material. Further inquiries can be directed to the corresponding author.

## Ethics statement

The studies involving humans were approved by Dokuz Eylül University Hospital and Ege University Hospital (Izmir, Turkey) and Pediatric Research Center, New Children’s Hospital, University of Helsinki, and Helsinki University Hospital (Helsinki, Finland) Ethics Committee decree number: Ethics Committee Approval HUS/2426/2018, Research Permit HUS/54/2019. The studies were conducted in accordance with the local legislation and institutional requirements. Written informed consent for participation in this study was provided by the participants’ legal guardians/next of kin.

## Author contributions

All the authors contributed to the research concept and design. AD, EB, SD, AA, and AB contributed to the collection and/or assembly of data. All the authors contributed to data analysis and interpretation. AD and MH contributed to writing the article. All authors contributed to the article and approved the submitted version. All the authors have accepted responsibility for the entire content of this submitted manuscript and approved the submission.
